# So alike yet so different. Differential expression of the long non-coding RNAs NORAD and HCG11 in breast cancer subtypes

**DOI:** 10.1590/1678-4685-GMB-2020-0153

**Published:** 2021-03-19

**Authors:** Carolina Mathias, Gabrielle Araújo Pedroso, Fernanda Rezende Pabst, Rubens Silveira de Lima, Flavia Kuroda, Iglenir João Cavalli, Jaqueline Carvalho de Oliveira, Enilze Maria de Souza Fonseca Ribeiro, Daniela Fiori Gradia

**Affiliations:** 1 Universidade Federal do Paraná, Departamento de Genética, Programa de Pós Graduação em Genética, Curitiba, PR, Brazil.; 2 Hospital Nossa Senhora das Graças, Centro de Doenças da Mama, Curitiba, PR, Brazil.

**Keywords:** Breast cancer, lncRNA, NORAD, HCG11, regulon

## Abstract

Breast cancer (BC) is a heterogeneous disease, and it is the leading cause of death among women. NORAD and HCG11 are highly similar lncRNAs that present binding sites for PUMILIO proteins. PUMILIO acts on hundreds of mRNA targets, contributing to the modulation of gene expression. We analyzed the expression levels of NORAD and HCG11 in the BC subtypes luminal A (LA) and basal-like (BL), and the regulatory networks associated with these lncRNAs. In the analysis of TCGA cohort (n=329) and Brazilian BC samples (n=44), NORAD was up-regulated in LA while HCG11 was up-regulated in BL subtype. An increased expression of NORAD is associated with reduced disease-free survival in basal-like patients (p = 0.002), which suggests that its prognostic value could be different in specific subtypes. The biological pathways observed for the HCG11 network are linked to the epithelial-to-mesenchymal transition; while NORAD associated pathways appear to be related to luminal epithelial cell transformation. NORAD and HCG11 regulons respectively present 36% and 21.5% of PUMILIO targets, which suggests that these lncRNAs act as a decoy for PUMILIO. These lncRNAs seem to work as players in the differentiation process that drives breast cells to acquire distinct phenotypes related to a specific BC subtype.

## Introduction

Since the advent of RNA-seq, the possibility to analyze large amounts of transcriptomic data from different types of organisms, tissues and cells generated lots of information thus demanding time and bioinformatics approaches to be better understood. One of the science fields that has significantly benefited from these data is cancer biology. The transcriptome of thousands of patients of different cancer types is now available in databases like The Cancer Genome Atlas (TCGA), and a refined analysis of these data is being performed by researchers around the world. 

The expression patterns of a specific class of transcripts named long non-coding RNAs (lncRNA) is drawing attention because of their importance in regulating physiological processes, controlling normal organogenesis, also for playing a role in tumor development (Fatica and Bozzoni, 2014; Yan *et al*., 2015; Melissari and Grote, 2016; Schmitz *et al*., 2016). LncRNAs are transcripts larger than 200 nucleotides in length that do not appear to have protein-coding potential, although some of those may produce small functional peptides (Cabili *et al*., 2011). The knowledge about the role of these molecules in cancer progression is continuously growing. Oncogenic and tumor suppressor activities have been spotted (Beckedorff *et al*., 2013), but there is information about no more than a few dozens of them (de Oliveira *et al*., 2019). Studies have found that lncRNA exhibited a tissue-specific pattern of expression greater than protein-coding genes, making them attractive as tissue-specific biomarkers (Cabili *et al*., 2011; Derrien *et al*., 2012). 

From the public health perspective, breast cancer is one of the most important types of cancer because of the social and economic impact caused by its high incidence (Bray *et al*., 2018), leading to the need for an urgent development of new diagnostic and therapeutic strategies. 

Breast cancer (BC) can be classified by means of molecular or immunohistochemical approaches. In both cases, the tumors are identified mainly through the expression of estrogen receptor (ER), progesterone receptor (PR), and Human Epidermal Growth Factor Receptor 2 (HER2), also known as ERBB2. Luminal A subtype express ER and PR does not express HER2; luminal B subtype is ER-positive, PR positive/negative, and HER2 positive/negative; HER2-enriched subtype overexpress HER2 and are ER/PR negative; while the molecular subtype basal-like or its immunohistochemical counterpart triple-negative, does not express either of them (Goldhirsch *et al*., 2013). Patients with luminal subtypes usually have a better prognosis than those with HER2-enriched or basal-like BC (Feng *et al*., 2018). 

As BC is a heterogeneous disease, the proper classification of the subtypes has allowed for improvement in the treatment of patients, with the use of specific drugs; even though for basal-like (the most aggressive type), there is no specific therapy, which leads to a worse outcome (Tang *et al*., 2016; Waks and Winer, 2019). In this way, there is an increasing need for new therapeutic targets, and lncRNAs may facilitate both the tumor classification and the identification of other pathways enrolled in breast carcinogenesis, considering the tissue-specific pattern of the expression exhibited by them.

NORAD (non-coding RNA activated by DNA damage) is a lncRNA located on chromosome 20q11.23, containing 5,339 nucleotides, only one exon, and its transcript is preferably located in the cytoplasm. This lncRNA has some particularities such as a high expression (500 -1,000 copies per cell), it is present across tissues and cell lines with comparable levels across most embryonic and adult tissues, and it also presents multiple binding sites for the RNA binding proteins Pumilio (Lee *et al*., 2016; Tichon *et al*., 2016).

HCG11 (HLA Complex Group 11) is also a lncRNA that presents 91% of identity to the NORAD sequence (Figure S2). Both NORAD and HCG11 are polyadenylated lncRNAs, and their genes appear to be transcribed from promoters derived from long terminal repeats of retroviruses (Bohn *et al*., 2018). HCG11 is located on chromosome 6p22.2, it has 5,696 nucleotides, and like NORAD, it has only one exon. The cellular location of this molecule can be either nuclear or cytoplasmic, depending on the cell type (Tichon *et al*., 2016). 

The high identity observed between NORAD and HCG11 lncRNAs keeps the Pumilio Recognition Elements (PRE) distributed along their sequences preserved. Considering the canonical binding sites, there are at least 15 and 16 PRE in NORAD and HCG11, respectively (Figure S2). Pumilio is an RNA binding protein known for repressing gene expression by binding in the 3’ untranslated region of their mRNA targets (Zamore *et al*., 1997; Wickens *et al*., 2002). NORAD was described as a regulator of Pumilio, by sequestering it, avoiding the degradation of its targets, but the molecular function of HCG11 has not been reported yet (Tichon *et al*., 2016).

Despite their similarity, the general expression of HCG11 is approximately 20-fold lower than the NORAD (Tichon *et al*., 2016), and very little is known about the HCG11 function. 

Here we performed a global expression analysis of the lncRNAs NORAD and HCG11 in the most contrasting BC subtypes - luminal A and basal-like, focusing on their regulatory networks, and managing to better distinguish of each distinct phenotype. 

## Subjects and Methods

### TCGA analysis

Information from 329 cases of invasive breast carcinoma (231 luminal A and 98 basal-like) were downloaded through CBioPortal platform (https://www.cbioportal.org). All RNA-seq data and clinical data are available in the TCGA database following ethics, laws, and policies from the program. Large-scale RNA-seq datasets from The Cancer Genome Atlas (TCGA) were assessed and analyzed by the open-access web resource “The Atlas of Noncoding RNAs in Cancer” (TANRIC) (Li J *et al*., 2015). Total RNA-seq data were extracted through XenaBrowser (https://xenabrowser.net/datapages/) of GDC TCGA Breast Cancer (BRCA) project.

### Sample collection

Forty-four breast tumor tissue specimens were obtained from patients during a surgical procedure at Hospital Nossa Senhora das Graças, Curitiba (PR), Brazil. The surgical samples are from different tumor types, according to origin, subtype, and location of the tumors. The selection of mammary tumor samples used in the present study was based on the tumor subtype and classified according to immunohistochemistry into four groups: HER2, luminal A, luminal B, and triple-negative (Goldhirsch *et al*., 2013). All specimens were maintained at RNA later solution (Thermo Fisher) and kept at -80 ºC for long term storage. Clinical and histopathologic characterization of the breast tumor tissue is represented in Table S1. 

This study was conducted by the guidelines for research involving human subjects and with the approval by Comissão Nacional de Ética em Pesquisa - CONEP Process No. 09718912.8.0000.0093). All participants signed informed written consent following the principles of the Declaration of Helsinki.

### RNA isolation and cDNA synthesis:

Total RNA was isolated from tumor tissues using Total RNA Purification Kit (RNAeasy QIAGEN) according to the manufacturer’s instructions. RNA quantification was performed on a spectrophotometer (Nanodrop Spectrophotometer ND-100, Thermo Scientific, Wilmington, USA). The degree of purity of the preparation was estimated by the relation Abs260nm / Abs280nm, considering, as good purity, a ratio close to 2.0. Samples of cDNA were synthesized from 0.5 μg of tumor RNA using the SuperScript III enzyme (Invitrogen) and random primers, and following the manufacturer’s instructions. To ensure the absence of genomic DNA contaminants in samples, a DNase (QIAGEN) treatment was performed, in addition to a control reaction of cDNA synthesis, without the addition of the enzyme reverse transcriptase according to the manufacturer’s guidelines. 

### Quantitative real-time PCR

Real-time PCR was performed by using the Viia-7 Sequence Detection Systems (Applied Biosystems). The primer sequences used were as follows NORAD: F: 5’TGATAGGATACATCTTGGACATGGA3’ R: 5’ AACCTAATGAACAAGTCCTGACATACA3’; HCG11: F: 5′- AGGAGTGGTTGCATTTGGGA-3′ R: 5′-CCCACCACGCAGTGAATAG-3’ GUSβ: F: 5’- CCGAGTGAAGATCCCCTTTTTA-3’ R: 5’-GAAAATATGTGGTTGGAGAGCTCATT-3’. PCR was performed in a total volume of 10 μl containing 4.5 μlcDNA template, 5 μl2 X SYBR® Green PCR Master Mix (Applied Biosystems; Darmstadt, Germany), and 0.5 μl of solution that contains forward and reverse primers. The following cycling conditions were used: initial activation at 95 °C for 10 min, denaturation at 95 °C for 15 s, and 60 °C for 60 s. The gene expression was determined by using the 2^-ΔΔCt^ method (Livak and Schmittgen, 2001).

### Statistical analysis

An analysis of the Student's t-test was used to examine the NORAD and the HCG11 expression between luminal A and basal-like subtypes.

For the survival analysis, patients were stratified into values below and above the median and the quartiles (P25 and P75). The Kaplan-Meier curves (method) and log-rank tests were used to estimate overall survival, which was calculated to the last follow-up, or death event. Data were analyzed statistically through GraphPad Prism 7 and R Studio software.

### Reconstruction of regulons

RNA-seq data from the TCGA cohort was used to reconstruct regulatory networks considering NORAD and HCG11 as the central regulators of the network. The reconstruction was performed through the RTN package (http://bioconductor.org/packages/release/bioc/html/RTN.html). This package provides classes and methods for transcriptional network inference and analysis using conditional mutual information. The list of genes in each regulon is shown in Table S2. 

After the reconstruction of the regulons, we investigated their activity in luminal A and basal-like subtypes, by using the RTN Survival Package (http://bioconductor.org/packages/release/bioc/html/RTNsurvival.html). We generated the Differential Enrichment Score (dES) plot to visualize regulon activity in the subtypes, assessing the stratification of a cohort based on the regulon activity. As a result, we have a network of co-expression formulated from the data of mutual information from potential genes positively or negatively modulated, in agreement with HCG11 or NORAD. Enrichment analysis was performed by using FunRich (Version 3.1.3) software (Pathan *et al*., 2015), to evaluate the biological process involved in each regulon. From data deposited on the MSigDB platform, we extracted molecular signatures for the luminal A and basal-like subtypes (https://www.gsea-msigdb.org/gsea/msigdb). We integrated the data of the reconstructed regulons for NORAD and HCG11 to identify overlaps through" Fisher’s exact test.

### Chromatin state analysis

TCGA cohort data from promoter accessibility and methylation were extracted from Xena Browser (Available at https://xenabrowser.net/datapages/?hub=https://atacseq.xenahubs.net:443). In total, there were 18 Luminal A and 15 Basal-like samples. All these samples were the same for promoter accessibility and methylation (Corces *et al*., 2018).

Promoter accessibility was evaluated through ATAC-seq and methylation using Illumina Human Methylation 450k. The chromatin accessibility information analyzed was made by taking the promoter region as a parameter. For this region, the probe used was BRCA 69522.

All data generated for this study are included in this article (and its supplementary information files).

## Results

### The lncRNAs NORAD and HCG11 are differentially expressed among breast cancer subtypes

**Figure 1:**
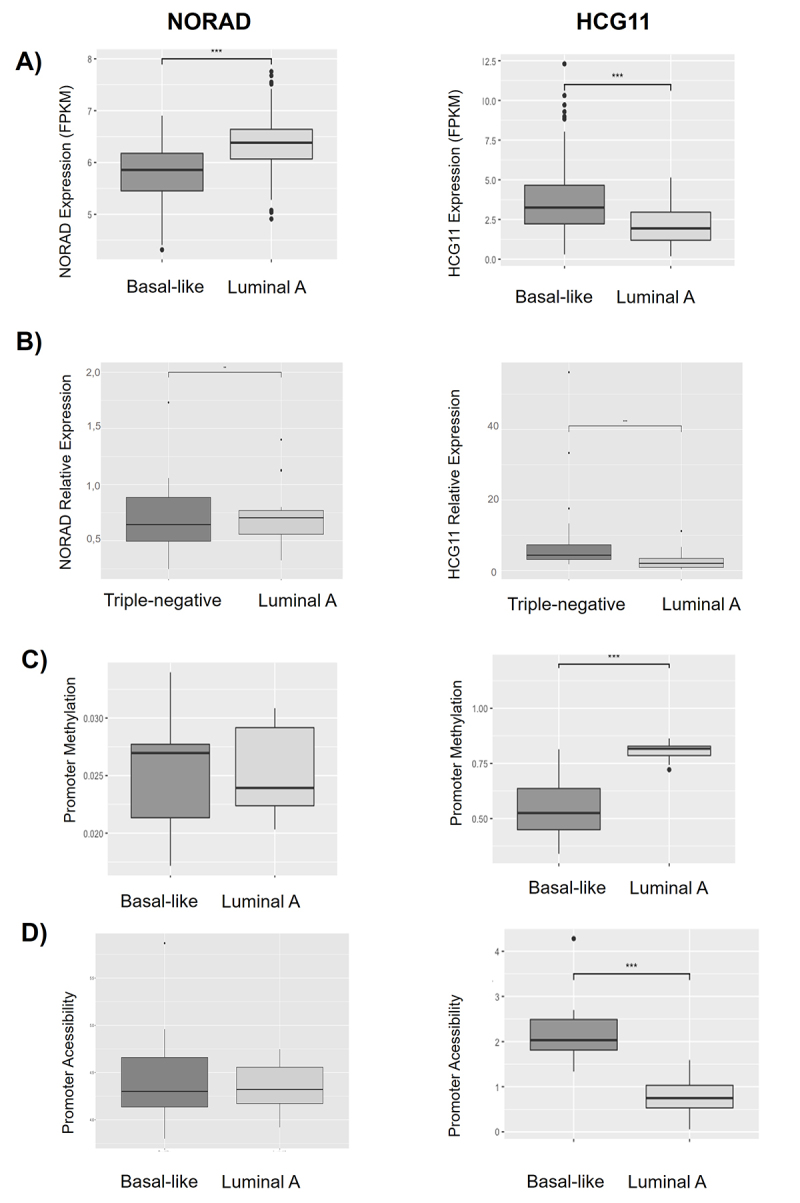
NORAD and HCG11 presented different expression patterns in BC subtypes. (A) Analyzing TCGA cohort on luminal A (n = 231) and basal-like (n = 98) subtypes, NORAD was upregulated in Luminal A while HCG11 was upregulated in basal-like subtype. (B) Relative expression measured by RT-qPCR on luminal A (n = 21) and triple-negative (n = 23) Brazilian BC samples, NORAD and HCG11 maintained the pattern of expression observed in TCGA cohort. (C) TCGA cohort data for promoter methylation. HCG11 presented in Luminal A subtype greater promoter methylation. (D) TCGA cohort data for promoter accessibility was performed by assay for transposase-accessible chromatin with high-throughput sequencing (ATAC-seq). HCG11 exhibited a more accessible promoter in Basal-like subtype. * p < 0.05; ** p < 0.01; *** p < 0.001; N.S - Not Significative

To investigate the expression pattern of NORAD and HCG11 in BC patients, we examined the RNA-seq data from the TCGA datasets on luminal A (n = 231) and basal-like (n = 98) subtypes. NORAD is less expressed in patients that belong to the poorest prognostic subtype basal-like in comparison to luminal A patients (p < 0.001). From the same cohort, analysis of HCG11 expression showed an opposite outcome, and higher levels were observed in basal-like patients (Figure 1A) compared to luminal A patients (P < 0.001). These differences were confirmed in an independent cohort from Brazilian patients classified by immunohistochemistry, including luminal A (*n =* 21) and triple-negative (*n =* 23) tumor samples, classified by RT-qPCR (Figure 1B).

### The expression regulation of the lncRNAs NORAD and HCG11 seems to be given by different molecular mechanisms

In order to raise more information about NORAD and HCG11 expression regulation, we extracted cohort information about promoter accessibility and methylation from TCGA. As expected for the expression data, HCG11 promoter is significantly more accessible and also less methylated in basal-like patients when compared with the luminal A ones. Similar observations were not found on NORAD promoter. Interestingly, for this lncRNA, there is no difference for promoter accessibility or methylation between the two BC subtypes studied (Figure 1C and 1D). 

These results bring evidence that NORAD and HCG11 have different regulation profiles, which may be related to the differential expression profile in BC subtypes.

### NORAD expression has an impact on survival in Basal-like breast cancer 

Considering the expression differences of NORAD and HCG11 in BC subtypes, we decided to investigate its prognostic value using survival data available from TCGA cohort. The median and quartile values were used as cut-offs for the groups (P25 and P75). Survival analysis for both NORAD and HCG11 in the entire cohort did not show statistically significant differences (Figure S1).

**Figure 2:**
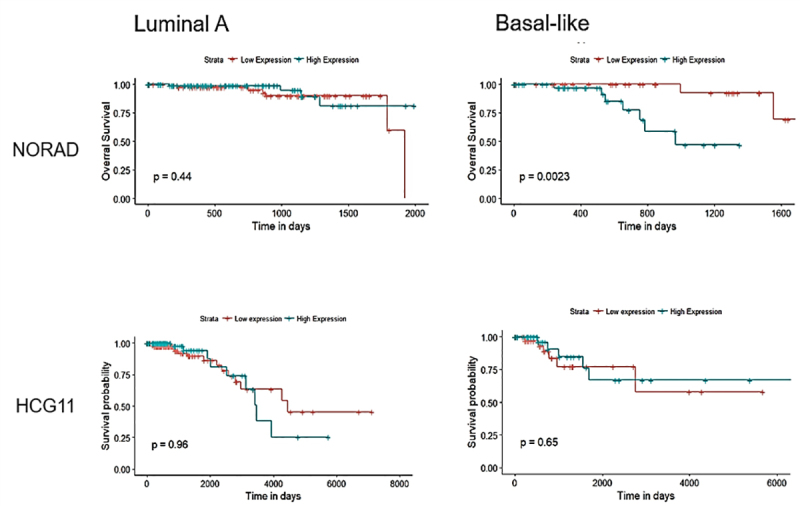
Overall survival determined by the Kaplan-Meier plots method and the log-rank test according to NORAD and HCG11 expression (median value). Red and blue lines refer to patients with low and high expression, respectively. Disease-free survival information of breast cancer patients was downloaded from the TCGA cohort.

We found that an increased expression of NORAD is associated with reduced disease-free survival only in basal-like patients (P75, p = 0.002), suggesting that the prognostic value of NORAD could be different in specific subtypes. For HCG11, differences in expression do not correlate with overall survival (OS) in neither studied group of patients (Figure 2). 

### NORAD and HCG11 present independent regulons

**Figure 3:**
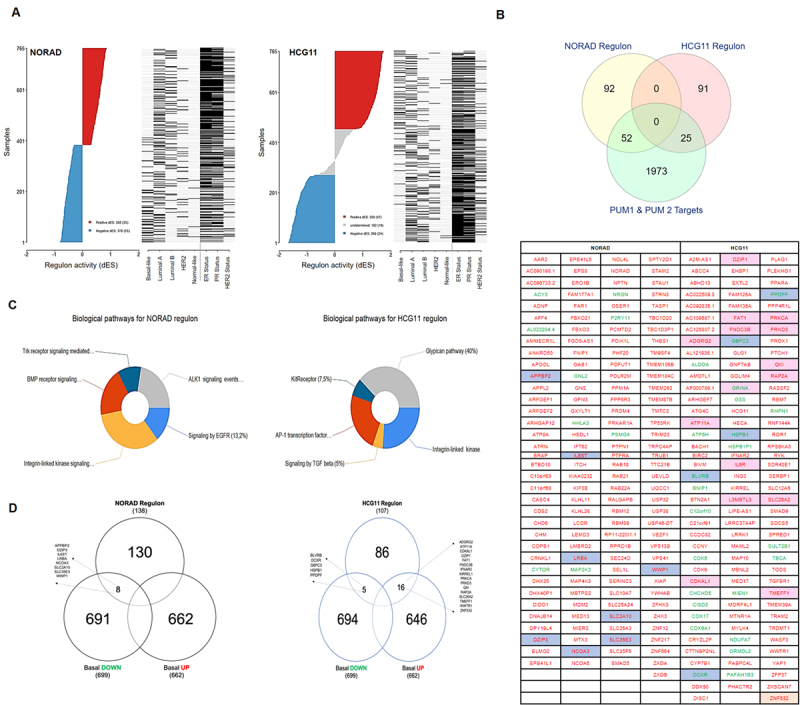
Ranked dES plot for NORAD and HCG11 using TCGA data. (A) Stratification of TCGA cohort-based on regulon activity and status of the key attributes: ER, PR, and HER2 status. The red section of the plot indicates regulon activation, and the blue section regulon repression. (B) Venn diagram showing the intersection among NORAD and HCG11 regulons and the potential PUMILIO targets; gene list is representing the gene set from both regulons. Genes that present positive or negative mutual information in each regulon are shown in red or green, respectively. Those that are also up or down-regulated in the basal-like signature are shown in blue or pink cells, respectively. (C) Gene Ontology (GO) analysis of the HCG11 and NORAD regulons indicated in (B). (D) Comparison among molecular signatures for the basal-like (up and down-regulated protein-coding genes) with regulons for NORAD and HCG11.

After observing that NORAD and HCG11 have a particular expression pattern for each BC subtype, we performed a reconstruction of the transcriptional network based on mutual information and all potential targets using TCGA cohort transcriptomic data. This strategy allowed us to generate the regulons coordinated by these lncRNAs, in BC subtypes and also according to the receptor status (Castro *et al*., 2016). The networks comprised 145 and 116 genes, respectively, for NORAD and HCG11 regulons (Figure 3B and Table S2). The profile of each regulon focuses on hormone status (ER + and PR +), and it shows a positive activity for NORAD but a negative activity for HCG11. This status is well observed in basal-like samples, where the HCG11 regulon is active while it is inactive in NORAD (Figure 3A).

Molecular signatures for the luminal A and basal-like subtypes were compared with HCG11 and NORAD regulons. This signature showed up and down regulated genes in the molecular subtypes of the disease. No list overlaps were found when comparing the molecular signature of the luminal A subtype to the other regulons analyzed. Through a Fisher’s exact test, we observed a significant relationship between the two classification factors HCG11 and NORAD regulons, and also basal-like genes-up and basal-like genes-down categories (p <0.01).

Interestingly, these two networks do not share common elements (Figure 3B). To better understand the biological processes underneath each regulon, we performed Gene Ontology (GO) analysis with the involved genes. No significant p-values (BH correction) of the enrichment terms were obtained from both GO analyses. Nevertheless, the set of biological pathways observed for the HCG11 network are closely linked to epithelial-to-mesenchymal transition (EMT), a crucial mechanism for malignant phenotype tumor acquiring (Felipe Lima *et al*., 2016). On the other hand, NORAD associated biological pathways, include BMP and ALK1 signaling, that are related to luminal epithelial cell transformation (Figure 3B)(Chapellier and Maguer-Satta, 2016).

Within the set of genes belonging to the NORAD and HCG11 regulons, a significant portion (36% and 21.5%, respectively), are molecular targets of Pumilio. A differential expression analysis of the *PUM1* and *PUM2* mRNAs was performed, comparing the luminal A and basal-like samples of the TCGA cohort. No significant difference was observed (p <0.05) when comparing these two subtypes (data not shown). Considering only one (PSMG4), Pumilio targets presented a coordinated expression with NORAD or HCG11 regulons.

## Discussion

Breast cancer is a heterogeneous disease and, based on the molecular profile, can be classified in four main subtypes: luminal A and B, HER2-enriched, and basal-like (Sorlie *et al*., 2003; Heng *et al*., 2017). Using high-throughput transcriptome sequencing data from BC patients, we found that in addition to mRNAs, ncRNAs also show a differential expression profile when comparing normal and tumor tissues (Chan and Tay 2018). Differential expressions of many lncRNAs have been associated with BC (de Oliveira *et al*., 2019; Mathias *et al*., 2019).

NORAD is a lncRNA with some unusual aspects when compared with other molecules from the same class. It is conserved between organisms (Guttman *et al*., 2009) and highly expressed in many cell types (Tichon *et al*., 2016), which suggests an important role in the cell. The relationship between NORAD expression and tumor tissues was previously observed in different types of cancer, in which the poorest prognosis was associated not only when NORAD was up-regulated (Li H *et al*., 2017; Wu *et al*., 2017; Huo *et al*., 2018; Li Q *et al*., 2018; Zhang *et al*., 2018) but also when it was downregulated (Hu *et al*., 2017). HCG11 is a paralog of NORAD, and despite the high similarity between them, the molecular function of HCG11 is still unknown.

Here we investigated the expression profile of NORAD and HCG11 in tumor and non-tumor breast tissue RNA-seq datasets from TCGA, focusing on each breast cancer subtype and also in tumor samples from Brazilian patients.

A study published in 2016 (Liu *et al*., 2016) looking for lncRNAs as prognostic markers for human breast cancer has associated the poor overall survival (OS) with the expression levels (upregulation) of four lncRNAs, including NORAD (LINC00657) and HCG11. This study has used the same information from TCGA cohort but with no distinctions among the subtypes. In the same study, Liu *et al*. (2016) observed that NORAD and HCG11 upregulation were significantly associated with OS. When we split breast cancer patients into subtypes and evaluated the expression levels of each lncRNA, we could observe that a higher expression of NORAD was found in luminal subtypes, usually lower-risk patients, in comparison to basal-like, which have a higher risk of relapse. Similar data was seen in two other cohorts from the GSE42568 dataset (n = 104), and National Taiwan University (n = 90) (Tan *et al*., 2019). HCG11 shows the opposite pattern, with a higher expression in basal-like/triple-negative samples. Corroborating these findings by RT-qPCR, we observed a similar result in Brazilian breast cancer patients, and a recent study with 80 breast cancer patients showed that tumors with up-regulation of HCG11 were mostly ER-negative (Dashti *et al*., 2020). Those observations reinforce the heterogeneous nature of breast cancer, making it necessary to discriminate each subtype for proper disease evaluation. In this regard, lncRNAs expression studies may help for a better stratification of subtypes.

We also observed a significant relationship between the two regulons and basal-like genes-up as well as basal-like genes-down categories (p <0.01), which suggests their involvement in breast cancer molecular phenotype. The method used to compute the regulons potentially mediated by HCG11 and NORAD was previously used, considering transcription factors. We are proposing here the use of this method to obtain co-expression networks for the lncRNAs in question. Since little is known about the biology of these RNAs, it is hard to make a direct regulation inference; however, we can highlight a group of genes regulated positively or negatively with the lncRNAs in question. By investigating the prognostic significance of these lncRNA expressions in breast cancer subtypes, we found that an increased expression of NORAD is associated with reduced disease-free survival only in basal-like patients (P75, p *=* 0.002) (Figure 2). In general terms, basal-like phenotype presents a lower expression of NORAD than other subtypes, even though, in this group of patients, those that show a higher expression of NORAD have the poorest prognosis in the 2,000 days after diagnosis. When the overall survival of HCG11 in subtypes was analyzed, we did not find an association with this lncRNA expression. The discrepancy in our results and those made by Liu *et al*. (2016) may be a consequence of using distinct strategies of analysis. While they used an expression level of HCG11 higher than 2 (exp > 2) to select the patients, we chose for using the median and also the 25th and the 75th percentiles to define high and low expression groups. 

As it occurs with protein-coding genes, lncRNAs expressions can be controlled both transcriptionally (Bunch, 2018) and post-transcriptionally (Yamamura *et al*., 2018). To better understand NORAD and HCG11 regulation, we extracted cohort data of accessibility and methylation status at the lncRNAs promoter sequence from TCGA. The accessibility data was obtained by means of the the assay for transposase-accessible chromatin using sequencing (ATAC-seq); and the methylation data were generated by using Illumina Human Methylation 450k.

Considering the HCG11 promoter accessibility, a significative difference between luminal A and basal-like subtypes was observed, being more accessible in the basal-like subtype. In contrast, HCG11 promoter methylation was more evident in luminal A than in basal-like. These results match with the difference observed in the expression level of HCG11 in the subtypes, suggesting regulation at chromatin level of this lncRNA. In contrast, promoter accessibility or methylation differences were not observed in NORAD when luminal A and basal-like subtypes were compared. Even with no differences regarding accessibility, NORAD promoter in cancer samples does not seem exposed. Recently, it has been demonstrated that histone deacetylation and nucleosome occupancy by YAP, TAZ, TEAD, and NuRD complex, are responsible for NORAD expression regulation in breast cancer cell lineage (Tan *et al*., 2019). The information suggests a different mechanism of gene regulation at transcriptional level for these lncRNAs.

In order to understand the functions of these lncRNAs, analyses of NORAD sequence have shown the presence of multiple binding sites for the conserved RNA binding proteins Pumilio (Pum1/Pum2) (Lee *et al*., 2016; Tichon *et al*., 2016) and pull-down assays showed the interaction with RBMX (RNA Binding Motif Protein X-Linked) (Munschauer *et al*., 2018). The association of NORAD with these proteins reinforces the idea that this lncRNA may act as a scaffold for ribonucleoprotein complexes. As it occurs with NORAD, HCG11 has multiple binding sites for Pum proteins (Bohn *et al*., 2018). It has been shown that the interaction of NORAD with Pumilio promotes the molecular decoy of these proteins in cancer cells (Tichon *et al*.,2018; Wang *et al*., 2018).

Pumilio proteins (PUM) belong to the PUF family of RNA-binding proteins (RBPs) that dictate stem cell fate in invertebrates by inhibiting expression/translation of target mRNAs (Wickens *et al*., 2002). Humans present two paralogs, PUMILIO1 (PUM1) and PUMILIO2 (PUM2), which control sets of functionally related mRNAs that encode proteins involved in angiogenesis, neurogenesis, cell cycle and differentiation. PUM1/2 also contribute to the self-renewal of human embryonic stem cells (ESCs) (Lee *et al*., 2007; Leeb *et al*., 2014) and human spermatogonial stem cells (SSCs) (Moore *et al*., 2003) and have been implicated in stem cell physiology by negatively regulating genes involved in cell differentiation (Shigunov *et al*., 2012; Silva *et al*., 2020). 

The high identity observed between NORAD and HCG11 lncRNAs keeps the Pumilio Recognition Elements (PRE) present in their sequences. We speculate that with the variation in the NORAD and the HCG11 expression, the availability of PUM proteins could vary as well, leading to differences in the expression of the mRNAs regulated by these proteins. To find a relationship among the mRNAs that make up NORAD and HCG11 regulons in BC subtypes and mRNAs regulated by PUMILIO proteins (Galgano *et al*., 2008), we compared those three groups of RNAs (Figure 3B). When looking at NORAD regulon, we observe that 36% (52 genes) are PUMILIO targets. With only one exception (PSMG4), these genes present a coordinated expression with NORAD. In the HCG11 regulon, 21.5% (25 genes) are PUMILIO targets and all of them have a coordinated expression with HCG11. As suggested before for NORAD (Lee *et al*., 2016; Tichon *et al*., 2016), we believe that these lncRNAs are acting as a decoy for PUMILIO, interfering in the expression of their targets in breast cancer.

Based on the paper that identified the NORAD interactome (Munschauer *et al*., 2018), and observing that a significant portion of the genes present in both regulons are not Pumilio targets, we believe that the expression fluctuations of these lncRNAs also interfere in the assembly of other complexes, thus altering cell metabolism via different mechanisms. For example, in glioma cells, it has been shown that the association of NORAD and AKR1B1 activates the ERK pathway, promoting a malignant phenotype (Luo *et al*., 2020). In an opposite fashion, NORAD works as a tumor suppressor by binding FUBP1 and promoting cell apoptosis in endometrial cancer (Han *et al*., 2020). NORAD also serves as a platform for S100P binding, suppressing lung and breast cancer metastasis (Tan *et al*., 2019). 

The regulatory networks controlled by NORAD and HCG11, though showing a set of genes that is not big enough to result in significant functional enrichment, indicate that essential processes in cancer signaling are present. In basal-like carcinomas, dedifferentiation is an important feature, as it leads tumor to become more aggressive (Felipe Lima *et al*., 2016). The transition from epithelial-to-mesenchymal phenotype (EMT) allows invasion and metastasis (Ye *et al*., 2017). The main endogenous EMT pathways include TGF-β, Notch, Wnt, Hedgehog, and receptor tyrosine kinases. The exogenous signals come from the extracellular matrix that activates the endogenous pathways (Fedele *et al*., 2017). HCG11 network is more active in basal-like subtype and includes genes that comprehend pathways related to EMT: TGF-β, glypican signaling, AP1 (Bakiri *et al*., 2015), suggesting the involvement of this lncRNA in malignant progression. Future studies are needed to understand the mechanisms of such observations.

Even with their great identity, NORAD and HCG11 seem to be involved in distinct cellular processes. The coexistence of two genes highly similar in their sequences, but with different expression patterns and functions, is also seen in coding genes. One example is acetylcholinesterase (AChE) and butyrylcholinesterase (BChE) genes. In humans, they share 53% of identity and a common precursor, but they are differently expressed both temporally and spatially (Massoulié *et al*., 1993).

## Conclusions 

Our findings indicate that NORAD and HCG11 are differentially expressed in breast cancer subtypes and participate in distinct regulatory networks. We postulate that these lncRNAs are regulated by specific transcriptional mechanisms, and that they may be essential players in triggering off breast cancer subtypes by regulating mRNAs availability and by acting as a scaffold for RNA-protein complexes in the cell.

## Supplementary material

The following online material is available for this article:

Table S1 - Clinical-pathological parameters of patients.

Table S2 - Regulons reconstruction. List of genes.

Figure S1 - CLUSTAL sequence alignment of NORAD and HCG11.

Figure S2 - Kaplan-Meier plots representing TCGA BRCA entire cohort stratified by NORAD and HCG11 median expression.

## References

[B1] Bakiri L, Macho-Maschler S, Custic I, Niemiec J, Guío-Carrión A, Hasenfuss SC, Eger A, Müller M, Beug H, Wagner EF (2015). Fra-1/AP-1 induces EMT in mammary epithelial cells by modulating Zeb1/2 and TGFβ expression. Cell Death Differ.

[B2] Beckedorff FC, Amaral MS, Deocesano-Pereira C, Verjovski-Almeida S (2013). Long non-coding RNAs and their implications in cancer epigenetics. Biosci Rep.

[B3] Bohn JA, Van Etten JL, Schagat TL, Bowman BM, McEachin RC, Freddolino PL, Goldstrohm AC (2018). Identification of diverse target RNAs that are functionally regulated by human Pumilio proteins. Nucleic Acids Res.

[B4] Bray F, Ferlay J, Soerjomataram I, Siegel RL, Torre LA, Jemal A (2018). Global cancer statistics 2018: GLOBOCAN estimates of incidence and mortality worldwide for 36 cancers in 185 countries. CA Cancer J Clin.

[B5] Bunch H (2018). Gene regulation of mammalian long non-coding RNA. Mol Genet Genomics.

[B6] Cabili MN, Trapnell C, Goff L, Koziol M, Tazon-Vega B, Regev A, Rinn JL (2011). Integrative annotation of human large intergenic noncoding RNAs reveals global properties and specific subclasses. Genes Dev.

[B7] Castro MA, de Santiago I, Campbell TM, Vaughn C, Hickey TE, Ross E, Tilley WD, Markowetz F, Ponder BA, Meyer KB (2016). Regulators of genetic risk of breast cancer identified by integrative network analysis. Nat Genet.

[B8] Chan JJ, Tay Y (2018). Noncoding RNA:RNA regulatory networks in cancer. Int J Mol Sci.

[B9] Chapellier M, Maguer-Satta V (2016). BMP2, a key to uncover luminal breast cancer origin linked to pollutant effects on epithelial stem cells niche. Mol Cell Oncol.

[B10] Corces MR, Granja JM, Shams S, Louie BH, Seoane JA, Zhou W, Silva TC, Groeneveld C, Wong CK, Cho SW (2018). The chromatin accessibility landscape of primary human cancers. Science.

[B11] Dashti S, Taherian-Esfahani Z, Kholghi-Oskooei V, Noroozi R, Arsang-Jang S, Ghafouri-Fard S, Taheri M (2020). In silico identification of MAPK14-related lncRNAs and assessment of their expression in breast cancer samples. Sci Rep.

[B12] de Oliveira JC, Oliveira LC, Mathias C, Pedroso GA, Lemos DS, Salviano-Silva A, Jucoski TS, Lobo-Alves SC, Zambalde EP, Cipolla GA (2019). Long non-coding RNAs in cancer: Another layer of complexity. J Gene Med.

[B13] Derrien T, Johnson R, Bussotti G, Tanzer A, Djebali S, Tilgner H, Guernec G, Martin D, Merkel A, Knowles DG (2012). The GENCODE v7 catalog of human long noncoding RNAs: analysis of their gene structure, evolution, and expression. Genome Res.

[B14] Fatica A, Bozzoni I (2014). Long non-coding RNAs: new players in cell differentiation and development. Nat Rev Genet.

[B15] Fedele M, Cerchia L, Chiappetta G (2017). The Epithelial-to-Mesenchymal transition in breast cancer: Focus on basal-like carcinomas. Cancers (Basel).

[B16] Felipe Lima J, Nofech-Mozes S, Bayani J, Bartlett JM (2016). EMT in breast Carcinoma-A review. J Clin Med.

[B17] Feng Y, Spezia M, Huang S, Yuan C, Zeng Z, Zhang L, Ji X, Liu W, Huang B, Luo W (2018). Breast cancer development and progression: Risk factors, cancer stem cells, signaling pathways, genomics, and molecular pathogenesis. Genes Dis.

[B18] Galgano A, Forrer M, Jaskiewicz L, Kanitz A, Zavolan M, Gerber AP (2008). Comparative analysis of mRNA targets for human PUF-family proteins suggests extensive interaction with the miRNA regulatory system. PLoS One.

[B19] Goldhirsch A, Winer EP, Coates AS, Gelber RD, Piccart-Gebhart M, Thürlimann B, Senn HJ, members P (2013). Personalizing the treatment of women with early breast cancer: Highlights of the St Gallen international expert consensus on the primary therapy of early breast Cancer 2013. Ann Oncol.

[B20] Guttman M, Amit I, Garber M, French C, Lin MF, Feldser D, Huarte M, Zuk O, Carey BW, Cassady JP (2009). Chromatin signature reveals over a thousand highly conserved large non-coding RNAs in mammals. Nature.

[B21] Han T, Wu Y, Hu X, Chen Y, Jia W, He Q, Bian Y, Wang M, Guo X, Kang J (2020). NORAD orchestrates endometrial cancer progression by sequestering FUBP1 nuclear localization to promote cell apoptosis. Cell Death Dis.

[B22] Heng YJ, Lester SC, Tse GM, Factor RE, Allison KH, Collins LC, Chen YY, Jensen KC, Johnson NB, Jeong JC (2017). The molecular basis of breast cancer pathological phenotypes. J Pathol.

[B23] Hu B, Cai H, Zheng R, Yang S, Zhou Z, Tu J (2017). Long non-coding RNA 657 suppresses hepatocellular carcinoma cell growth by acting as a molecular sponge of miR-106a-5p to regulate PTEN expression. Int J Biochem Cell Biol.

[B24] Huo H, Tian J, Wang R, Li Y, Qu C, Wang N (2018). Long non-coding RNA NORAD upregulate SIP1 expression to promote cell proliferation and invasion in cervical cancer. Biomed Pharmacother.

[B25] Lee MH, Hook B, Pan G, Kershner AM, Merritt C, Seydoux G, Thomson JA, Wickens M, Kimble J (2007). Conserved regulation of MAP kinase expression by PUF RNA-binding proteins. PLoS Genet.

[B26] Lee S, Kopp F, Chang TC, Sataluri A, Chen B, Sivakumar S, Yu H, Xie Y, Mendell JT (2016). Noncoding RNA NORAD Regulates Genomic Stability by Sequestering PUMILIO Proteins. Cell.

[B27] Leeb M, Dietmann S, Paramor M, Niwa H, Smith A (2014). Genetic exploration of the exit from self-renewal using haploid embryonic stem cells. Cell Stem Cell.

[B28] Li H, Wang X, Wen C, Huo Z, Wang W, Zhan Q, Cheng D, Chen H, Deng X, Peng C (2017). Long noncoding RNA NORAD, a novel competing endogenous RNA, enhances the hypoxia-induced epithelial-mesenchymal transition to promote metastasis in pancreatic cancer. Mol Cancer.

[B29] Li J, Han L, Roebuck P, Diao L, Liu L, Yuan Y, Weinstein JN, Liang H (2015). TANRIC: An interactive open platform to explore the function of lncRNAs in cancer. Cancer Res.

[B30] Li Q, Li C, Chen J, Liu P, Cui Y, Zhou X, Li H, Zu X (2018). High expression of long noncoding RNA NORAD indicates a poor prognosis and promotes clinical progression and metastasis in bladder cancer. Urol Oncol.

[B31] Liu H, Li J, Koirala P, Ding X, Chen B, Wang Y, Wang Z, Wang C, Zhang X, Mo YY (2016). Long non-coding RNAs as prognostic markers in human breast cancer. Oncotarget.

[B32] Livak KJ, Schmittgen TD (2001). Analysis of relative gene expression data using real-time quantitative PCR and the 2(-Delta Delta C(T)) method. Methods.

[B33] Luo L, Chen C, He H, Cai M, Ling C (2020). Silencing of Long Non-Coding RNA (LncRNA) Non-Coding RNA activated by DNA damage (NORAD) inhibits proliferation, invasion, migration, and promotes apoptosis of glioma cells via down regulating the xpression of AKR1B1. Med Sci Monit.

[B34] Massoulié J, Pezzementi L, Bon S, Krejci E, Vallette FM (1993). Molecular and cellular biology of cholinesterases. Prog Neurobiol.

[B35] Mathias C, Zambalde EP, Rask P, Gradia DF, de Oliveira JC (2019). Long non-coding RNAs differential expression in breast cancer subtypes: What do we know?. Clin Genet.

[B36] Melissari MT, Grote P (2016). Roles for long non-coding RNAs in physiology and disease. Pflugers Arch.

[B37] Moore FL, Jaruzelska J, Fox MS, Urano J, Firpo MT, Turek PJ, Dorfman DM, Pera RA (2003). Human Pumilio-2 is expressed in embryonic stem cells and germ cells and interacts with DAZ (Deleted in AZoospermia) and DAZ-like proteins. Proc Natl Acad Sci U S A.

[B38] Munschauer M, Nguyen CT, Sirokman K, Hartigan CR, Hogstrom L, Engreitz JM, Ulirsch JC, Fulco CP, Subramanian V, Chen J (2018). The NORAD lncRNA assembles a topoisomerase complex critical for genome stability. Nature.

[B39] Pathan M, Keerthikumar S, Ang CS, Gangoda L, Quek CY, Williamson NA, Mouradov D, Sieber OM, Simpson RJ, Salim A (2015). FunRich: An open access standalone functional enrichment and interaction network analysis tool. Proteomics.

[B40] Schmitz SU, Grote P, Herrmann BG (2016). Mechanisms of long noncoding RNA function in development and disease. Cell Mol Life Sci.

[B41] Shigunov P, Sotelo-Silveira J, Kuligovski C, de Aguiar AM, Rebelatto CK, Moutinho JA, Brofman PS, Krieger MA, Goldenberg S, Munroe D (2012). PUMILIO-2 is involved in the positive regulation of cellular proliferation in human adipose-derived stem cells. Stem Cells Dev.

[B42] Silva ILZ, Robert AW, Cabo GC, Spangenberg L, Stimamiglio MA, Dallagiovanna B, Gradia DF, Shigunov P (2020). Effects of PUMILIO1 and PUMILIO2 knockdown on cardiomyogenic differentiation of human embryonic stem cells culture. PLoS One.

[B43] Sorlie T, Tibshirani R, Parker J, Hastie T, Marron JS, Nobel A, Deng S, Johnsen H, Pesich R, Geisler S (2003). Repeated observation of breast tumor subtypes in independent gene expression data sets. Proc Natl Acad Sci U S A.

[B44] Tan B-S, Yang M-C, Singh S, Chou Y-C, Chen H-Y, Wang M-Y, Wang Y-C, Chen R-H (2019). LncRNA NORAD is repressed by the YAP pathway and suppresses lung and breast cancer metastasis by sequestering S100P. Oncogene.

[B45] Tang Y, Wang Y, Kiani MF, Wang B (2016). Classification, treatment strategy, and associated drug resistance in breast cancer. Clin Breast Cancer.

[B46] Tichon A, Gil N, Lubelsky Y, Havkin Solomon T, Lemze D, Itzkovitz S, Stern-Ginossar N, Ulitsky I (2016). A conserved abundant cytoplasmic long noncoding RNA modulates repression by Pumilio proteins in human cells. Nat Commun.

[B47] Tichon A, Perry RB, Stojic L, Ulitsky I (2018). SAM68 is required for regulation of Pumilio by the NORAD long noncoding RNA. Genes Dev.

[B48] Waks AG, Winer EP (2019). Breast cancer treatment: A review. JAMA.

[B49] Wang L, Du L, Duan W, Yan S, Xie Y, Wang C (2018). Overexpression of long noncoding RNA NORAD in colorectal cancer associates with tumor progression. Onco Targets Ther.

[B50] Wickens M, Bernstein DS, Kimble J, Parker R (2002). A PUF family portrait: 3’UTR regulation as a way of life. Trends Genet.

[B51] Wu X, Lim ZF, Li Z, Gu L, Ma W, Zhou Q, Su H, Wang X, Yang X, Zhang Z (2017). NORAD Expression is associated with adverse prognosis in esophageal squamous cell carcinoma. Oncol Res Treat.

[B52] Yamamura S, Imai-Sumida M, Tanaka Y, Dahiya R (2018). Interaction and cross-talk between non-coding RNAs. Cell Mol Life Sci.

[B53] Yan X, Hu Z, Feng Y, Hu X, Yuan J, Zhao SD, Zhang Y, Yang L, Shan W, He Q (2015). Comprehensive genomic characterization of Long Non-coding RNAs across human cancers. Cancer Cell.

[B54] Ye X, Brabletz T, Kang Y, Longmore GD, Nieto MA, Stanger BZ, Yang J, Weinberg RA (2017). Upholding a role for EMT in breast cancer metastasis. Nature.

[B55] Zamore PD, Williamson JR, Lehmann R (1997). The Pumilio protein binds RNA through a conserved domain that defines a new class of RNA-binding proteins. RNA.

[B56] Zhang J, Li XY, Hu P, Ding YS (2018). LncRNA NORAD contributes to colorectal cancer progression by inhibition of miR-202-5p. Oncol Res.

