# Supplementary material for: So alike yet so different. Differential expression of the long non-coding RNAs NORAD and HCG11 in breast cancer subtypes
Source: Genet Mol Biol. 2021 Mar 19;44(1):e20200153. doi: 10.1590/1678-4685-GMB-2020-0153 (PMC7976429; doi:10.1590/1678-4685-GMB-2020-0153)
Supplement: Table S1 - Clinical-pathological parameters of patients. [file 1415-4757-GMB-44-1-e20200153-s1.pdf]

Genetics and Molecular Biology

https://doi.org/10.1590/1678-4685-GMB-2020-0153

Supplementary Material to "So alike yet so different. Differential expression of the long non-coding RNAs NORAD and HCG11 in breast cancer subtypes."

Figure S1 - CLUSTAL sequence alignment of NORAD and HCG11. The Pumilio Recognition Element (PRE) sequences are highlighted in blue.

CLUSTAL sequence alignment of NORAD and HCG11 by Kalign (2.0)

```
NORAD   ---------AGTTCCGGTCCGGCAGAGATCGCGGAGAGACGCAGAACGCAGCCCGCTCCT
HCG11   CACTGCAAGAGTTCCGGTCTGGCAGAGAGCGCGGAGAGACGCAGAGCGCGGGCCGCTCCT

NORAD   CCAGGGCCCTCCAGGCCCTCCGGCCCCGGGCCGGCGGGTGAACTGGGGGGC--CCCGGGA
HCG11   CCAGGGCGCTCCAGGCCCTCCGGCCCCGGGTCGGCGGGTGAGCTAGGGGGCGCCCCGGGA

NORAD   CAGGCCGAGCCCTCTGCCCTGCAGATAACGGAGGCCTCTGCTGTGGCTGCCCACTGGCTG
HCG11   CAGGCCGCGCCCTCTGCCCTGCAGATACCGGAGGCCTCTGCTGTGGCT------------

NORAD   TGCCCGCCCACTGGCTGTGCCCAGACCTTGAAGCCGCAGCGAACCTCTCTTTCCCACCCC
HCG11   -----GCCCACTGGCTGGGCCCAGGCCTTGAAGCGGTGGCGAACGTCTCTTCCCTACCCT

NORAD   ACCTCGGTGACTAATGGCGGCCGTGGCGTCTCCCAGCCCGGACCCCGCCGGCACCCGGGT
HCG11   ACCTCGGTGACTGATGGCGGCGGCGGCCTCTCCCAGTCCGGACCCTGCCGGCCGTCGGGT

NORAD   CTCCCGACCCAA---------GCCTCGACGAAACCCCCGCAGAGCCGCCGGGACGCAGCG
HCG11   CTCCCGGCCCAAGCCTGCCGGGCCTGGACGAAACCCCCGCCGAGCAGCCTGGACGCAGCG

NORAD   CCTTTGGGCGGCGCTGGGCGTGGTGGGCCGGGAAGTATGGCGGCAGCTCGAACGCCGCGC
HCG11   CCTTTGGGTGGCGGCGGGAGTAGCGTGCCGGGAAGCATGGCGGCCGCTCGAACGCCGCGC

NORAD   GGCGGAGGCCATTAAGGCGTGGACGGCCCGGGAAGGCGGCCTAGGGACGCAAGCAGGCTC
HCG11   GGCGGAGGGCGTTAGGGCATGGAGGGCCCGGGAAGGCGGCCTAGGGACGCAGGCAGGCTC

NORAD   GGCCGCCTCTTTAGGCCACGGAGCCGCGCAGATCCGGTTCCCGGGTGACCACTCTGTCGC
HCG11   GGCCGCCTCTTCAGGCCACGGAGCCGC-CAGATCTGGGTCCCGGGTGACCACTCTGTCGC

NORAD   CATTGGGCGAGACCTACCTAGTCCTGACGACAACGGACAAAGGCCTTAAGGGGCCTGGAA
HCG11   CATTGGGCGAGACCTACCTAGTCCTAACGACAACGGACAAAGGCCTTAACGGGCCTGGGA

NORAD   GGTGAGCGAAGTCCCGAACGACGACGGGTGGAACGGTTAGCGGCCATCGGGCGGTTGGTC
HCG11   GGTGAGCGAAGTCCCGAACGACGACGGGTGGAACGGTTAGCGGCGATCGGGCGGTTGGTC

NORAD   TTCATTCTACCAGACTTTGCTGTCGGAAGAGAGAAATGGTAGAATGACAGGCCACGTTTG
HCG11   TCCGTACTACCAGACCTTGCTGTCGGAAGAGACAAATGGTAGAATGACAGGCCACGTTTG

NORAD   GCCCGTTGGAAATGCCCACCACCCTCTGGGAAGATTTACTGGCCGTTTATGGAAGGCCTG
HCG11   GCCCGTTGGAAATGCCCTCCATCCTCTGGGAAGATTCACAGGCCGTTTACGGAAGGCCTG

NORAD   TGTATATAATATGAAAAAGCTGCTCTCAACTCCACCCCAACCTTTTAATAGAAAACATTT
HCG11   TGTATATAATATGAAAAAGCTGCTCTCAACTCCACCCCAACCTTTTAATAGAAAACATTT

NORAD   GTCACATCTAGCCCTTCTAGATGGAAAGAGGTTGCCGACGTATGATAAAATAGAGTTAGA
HCG11   GTCACATCTAGCCCTTCTAGATGGAAAGAGGTTGCCGACGTATGATA-----GAGTTAGA

NORAD   AAGTTACACATCTTGTAAATTCTCATTTGTTTAAAAGAAATCATAGAAAATACATGTCTT
HCG11   AAGTCACACATCTTGTAAATTCTCATTTGTTTAAAAGAAATCATAGAAAATACATGTCTT

NORAD   CTGGAGATGACTTTTGGAAATGGAGTTGTTAAGACGGCCTCTGGAAGCGATACGTCCACG
HCG11   CCGGAGTTGACTTTTGGAAACTGAGTTGTTA-GACGGCCTCTAGAAGCGATACGTTCACG
```

NORAD TTTGTTAAGTGGGTTAGATGACATGGAGCTGGAAGACCTGAGAAGGAAGAGAAGAAGGTT
HCG11 TTTGTTCAGTGGGTTAGATGACATGGAGCTCGAAGACCTGAGAAGGAAAAAAAGAAGGTT

NORAD CTATGCTAGACTGGTCATATTTAGAAGACATTTTCATATTCTATCCATTGTTTTGTGTGC
HCG11 CTATGCTAGACTGGTCATATTTAGAAGACATTTTCATATTCTATCCATTGTTTTGTGTGC

NORAD ATTTTATTCCTCACTACTGTGTATATAGTTGACAATGCTAAGCTTTTTTGAAATGTCTCT
HCG11 ATTCGCTTCCTCACTACTATGTATGTAGTTGACAATGCTAAGCTTTTTTGAAATGTCTAT

NORAD TCTTTTTAGATGTTCTGAAGTGCCTGATAT--GTTAAAATTAGAGGTAGCAAAATCACAT
HCG11 TCTTTTTAGATATTCTGAAGTGTCTGATATATGTTAAAATTAGAGGTAGTAAAACCACAT

NORAD TTTGTAAATACCTTTTTGTTACAATTCATAGGAAA--TATTTTTGGGGGGGAATGGCCAA
HCG11 TTTGTAAATATCTTTTTGTTACAATTCATAGAAATGTTGTTTTTTGGGGGGAATGGCCAA

NORAD ATCACCTGTTGAGTAATACTCATTGTGTTTGTGCAGTGGTTCAGGGGAGGAGAGAGGAGG
HCG11 ATCACCTGTTGAGTAATACTCATTGTGTTTGTGCAGTAGTTCAGGGGAGGAGAGAGGAGG

NORAD GGGAGGTGCAGAGAGCTCTATGCCATCCTGTTTACAGCGAGGCAAGATGAATCATTATGT
HCG11 GGGAGGTGCAGAGAGCTCTATGCCATCCTGCTTACAGCGAGGCAAGATGAATCACTATGT

NORAD CTGTGCATTTTGTTTTACTTATCTGTGTATATAGTGTACATAAAGGACAGACGAGTCCTA
HCG11 CTGTGCATTTTGTTT----TATCTATGTATATAATGTACATAAAGGACAAACGAGTCCTA
NORAD ATTGACAACATCTAGTCTTTCTGGATGTTAAAGAGGTTGCCAGTGTATGACAAAAGTAGA
HCG11 ATTTACAACATCTAGTCTTTCTGGATGTTAAAGAGGTTGCCAGTGTATAACAAAACTAGA

NORAD GTTAGTAAACTAATATATTTTGTACATTTTGTTTTACAAGTCCTAGGAAAGATTGTCTTC
HCG11 GTTAGTAAACTAATATATTTTGTACATTTTGTTTTACAAGTCCTAGGAAAGACTGTCTTC

NORAD TGAAAATTTGA-------TGTCTTCTGGGTTGATGGAGATGGGAAGGGTTCTAGGCCAGA
HCG11 TGAAAATTTGAGCATTCTTGCCCACTGGGTTGATGGAGATGGGAAGGGTTCTAGGCCAGA

NORAD ATGTTCACATTTGGAAGACTCTTTCAAATTATAACTGTTGTTACATGTTTGCAGTTTATT
HCG11 ATGTTCACATTTGGAAGACTCTTTCAAATTATAACTGTTGTTACATGTTTGCAGTTTATT

NORAD CAAGACTGCTGT----ATACATAGTAGACAAATTAACTCCTTACTTGAAACATCTAGTCT
HCG11 CGAGACTGCTGTGTACATACATAGTGGACAAATTAACTCCTTACTTGAAACATTTAGACT

NORAD ATCTAGATGTTTAGAAGTGCCCGATGTATGTTAAATGTATAGGTAGTAAAATACCACTTT
HCG11 ATCTAGATGTTTAGAAGTGCCCGATGTATATTAAATGTAGAGGTAGTAAAATACCAATTT

NORAD GTAAATATCTTTTTGCTAAAATTCATAGGAAATGCTTTTGGAAATTGAATTGTGAAGCCA
HCG11 GTAAATATCTTTTTGCTAAAATTCATAGGAAATACTTTTGGAAGTTGAATTGTGAAGCCA

NORAD CCTTTGTGAACAGTATAGTAATGTCTATACTTGTTCAATAGTTTAGAGGAGGTAGGAGGG
HCG11 CCTTTGTGAGCAGTATATTACTGTCTATACTTGCTCAATGGTTTAGAGGAGGTGGGAGGG

NORAD AAGAAATTGCAAAAGGTAATATTACTAGTGTGTTCATACTTGGACATTTTCAGACACCAT
HCG11 AAGAAATTGCAAAAGATAATA-TGCTAGTGTGTTCATACTTGGACATTTTCAGACACCAT

NORAD TTTTCTATATGTTTTGTGCATTTTGTTTTGCTCTGTATATAGTATATATAATGGACAAAT
HCG11 TTTTCTGTATGTTTTGTGCATTTTGTTTTGCTCTGTATATAGTGTATATAATGGACAAAT

NORAD AGTCCTAATTTTTCAACATCTAGTCTCTAGATGTTAAAGAGGTTGCCAGTGTATGACAAA
HCG11 AGTCTTAATTTTTTAACATCTAG---------------AGGTTGCCAGTGTATGACAAA

NORAD GGAGTAAAATTAGCATATTTTGTACACTTTGTGTTGAAATTCGTAGGAAAACTTGTCTTC
HCG11 GTAGTAAAATTAGCATATTTTGTATGCTTTGTGTTGAAATTCATAGGAAAACTTGTCTTC

NORAD TGTAA-AGACTTTTGCATAGGAATTTGTTTGACCATCTCTAAGCATTACACGTGCCTGTA
HCG11 TGTAATTGACTTTTGCATAGGAATTTGTTCAGCCATCTCTAAGCATTACACATGCGTGTA

NORAD CTTGTCCACTGGATTGAAGGCAGAGAAGGAAGGGAGGAGGGAATGATTCAAGGCCAAAAT
HCG11 CTTGTCCACTGAATTGAAGGCAGAGAAGGAAGAGAAGAGGGAATGATTCAAGGCCAAAAT

NORAD GGCCACATTTAGAAGATACCTCAGATGATAACCATTGTTATGTGTGTGCAATTTTATTTA
HCG11 GGTCACATTTAGAAGATACCTTAGATGATAACCATTGTTATGTGTGTGCAGTTTTATTTA

NORAD ACAGTGCTGTGTATGTGGTGGACAAGTTATATGAAATATCTAGTCTTTCTAGATATTTGG
HCG11 ACAGTGCCGTGTACATGGTGGACAGGC--TATGAAATATCTAGTCTT--TAGATATTTGG

NORAD AAGTGCTTGATGTATTTAAA---AGTGGTAGTAGAATAACACTTT--GTAAATAGCTTTT
HCG11 AAGTGCTTGATGTATTTTAAAGTAGTAGTAGTAGAATAACACTTTTTGTAAATAGCTTTT

NORAD AAAAACTGATGGGAAATGCTGTTTGGAAGTGGAATTGTTGAACCACCTGGGAGGTGGGAG

HCG11 AAAAACTGATGGGAAATGCTGTTTGGAAGTGGATTTGTTGAACCACCTGGGAGGTGGGAG

NORAD GGAAGAAATTGCAAATGGTGTTTTGCCATTGTTTATTAGAAAATTTCAGCTTAATCCATT
HCG11 GGAAAAAAATTGCAAAAGGTGTTTTGCCATTGTTTATTAGAAAATTTCAGCTTAATCCATT

NORAD GTGTATATGTTACATGCATTTCATTTAACTTTGCTATACTGTATATATTTGTATATATAAC
HCG11 GCCTATATGTTACAAGCATTTCATTTAACTTTGCTATACTGTATATATTGTGTATATACT

NORAD GGACAAATTAGTCCCGATTTTATAATATCTAGTCTCTAGATATTAAAGAGGTTGCCAATG
HCG11 GGACAAATGAGTCCTGATTTTATAATATCTAGTCTGTAGCTATTAAAGAGGTTGCCAGTG

NORAD TATGACAGAAGTAGAGTTAGTAAACTAACACATTTTGTACACTTTGTTAAAATTTGTAGA
HCG11 TATGACAAAAGTA--GTTAGTAAACTAACGCATTTTGTACACTTTGTTAAAATTCATAGA

NORAD AAGGCTGTCTTCTGAAAAGGAC-TTTTGGAAGTGA---GATAACATCAGCTCTAAGTGAC
HCG11 GAGGCTGTCTTCTGAAAAGGACTTTTTGGAAGTGAAATGATAACATCAGCTCGAAGTGAC

NORAD AC--GTGCCTATATCCATCAGGTTGGTGGTGGAGAGGAGTTGGAAGGAATGAAGGGTTCT
HCG11 ACATGTGCTTATATCCACCAGGTTGGTGGTGGAGAGGAGTTGGAAGGAATGAAGGGTTCT

NORAD AGACCAGAATGTTCGTATTTAGAAGACACTATCAGATATAACCATTGTTACATGTGTGTA
HCG11 AGACCAGAATGTTCCTATTTAGAAGACACTTTCAGATATAACCATTGTTACATGTGTGTA

NORAD GTTTATTCAACCCTACTGTGTATATAGCGGACAAACTTAAGTCCTTATTTGAAACATCTA
HCG11 GTTTATTCAACAGTGCTATGTATATAGTGGACACACTTAAGTCCTTATTTGAAATATCTA
NORAD GTCTTTCTAGATGTTTAGAAGTGCACAAAGTATGTTAAAAGTAGAGGTAGTAAATAACAC
HCG11 GTCTTTCTAGATGTTTAGAAGTGCACAAAGCATGTTAAAAGTAGAGGTAGTAAGTAACAC

NORAD ATTTTGTAGCTATCCTTTTGATATGAAATATTGTCTTGGAAATTGATCAATTCTCTGAGC
HCG11 ATTTTGTAGTTAT---TTTGATATGAAATATTGTCTTGGAAATTGATCAATTCTCTGAGA

NORAD AGTACCCATTTTGATATTTGTGCTGGTTCAGGGGGAAGGAGGAGCACAAAGTGCAAAGGG
HCG11 AGTACACGTTATGATATTTGTGCTGGTTCAGGGGGAAGAAGGAGCACAAAGTTCAAAGGG

NORAD CTTTCTACCAGTGTCCAGTGTGTTTATGAGGAGGCACATTGACCATTGTCCCTTATGTCT
HCG11 CTTTCTACCAGTGTCCAGTGTGTTTATGATGAGGCACATTGACCATTGTCCCTTATGTCT

NORAD GCATTTTCATTTACTGTGCTGTGTATATAGTGTATATAAGCGGACATAGGAGTCCTAATT
HCG11 GCATTTTCGTTTACTGTGCT---------GTGTATATAAGCAGACATAGGAGTCCTAATT

NORAD TACGTCTAGTCGATGTTAAAAAGGTTGCCAGTATATGACAAAAGTAGAATTAGTAAACTA
HCG11 TATATCTAGTCGATGTTAAAGAGGTTGCCAGTGTATGGCAAAAGTAGAGTTAGTAAACTA

NORAD CTACATTGAGTACACTTTGTGTTAAAATTCATAGGGAAGACTTCTTAAAAACA--AGTGA
HCG11 ATACATTGAGTACACTTTGTGTTAAAATTCATAAGGAACACTTCTTAAAAACAGAAGTAA

NORAD AATTGTTAAAACCCC-----CCCTAAGCATTACAGATGGCTTATAGCTGTCCACGGGGTT
HCG11 AATTGTTAAACGCCCCCCCGCCCCCAAGCATTACAGATGGCTTATAGCTGTCAACGGGGTT

NORAD GGTAGAGGTGGGAAAGGGAAGGGTTCTAGGCCAGAATGTTCCTATTTAGAAGACACTCAA
HCG11 GGTAGAGGTCAGAAAGGGAAGTGTTCTAGGCCAGAATGTTCCTATTTAGAAGACACTCAA

NORAD ATTACAGTCTGTGTTATGTATGTATACCATTTATTCAATGCTACTGTGTATATAATGGAA
HCG11 ATTATAGTCTATGTTATGTATATATGCCATTTATTCAATACTACTGTGTATATAATGGAA

NORAD AACTTAAGTCCAGTTTGAAACATCTAGTCTTTCTAGGTGTTTAAAAGTGTACAACGGCCT
HCG11 AACTTAAGTGCAGTTTGAAACATCTAGTCTTTCTAGGTGTTTAAAAGTGCACAATGGTCA

NORAD GTCGCAGTGGCGCATGCCTGTAATCCCAGCACTTTGGGAGGCCGAGGCAGGCGGATCACG
HCG11 GGTGCGGTGGCTCACTCCTGTAATCCTGGCACTTTGGGAGGCTGAGGTGGGCAGATCACG

NORAD AGGTCAAGAGATCAGGACCATCTTGGCCAACATGGTGAAACCCCATCTTTACTAAAAATA
HCG11 AAGTCAAGGGATCGAGACTATCTTGGCCAACATGGTGAAACCCCATCTCTACAAAAAATA

NORAD CAAAAATTAGCTGGTCGTGGTGGTGCCCACCTGTAGCCCCAGTTACTCGAGAGGCTGAGG
HCG11 CAAAAATTAGCTGGTCGTGGTGGCACATGCCTGTAGTCCCAGCTACTCGGGAGGCTGAGG

NORAD CAGGAGAATCGCTTGAACTTGGGAGGCGGAAGTTGCAGTGAGCCAAGATCGCACCACTGC
HCG11 CAGGAGAATCGCTTGAGCCCGGGAGACAGAGGTTGCAGTAAGCCGAGACCACACCACTGC

NORAD ACTCCAGCCTGGCGACAGAGCGAGGCTCCGTTTCAAAAAAAAAAG-----------TGCA
HCG11 ACTCCAGCATGGCGACAGAGCAAGACTCCATCTCAAAAAAAAAAAAAAAAAAAAAATTCCA

NORAD CAATGTAGGTTAACAGTAGAGGGCTTAAGTAACACCCCTCTAAGCATTTGTTTTCAGTAC
HCG11 CAACATAGGTTAAAAGTAGAGGGCTAAAGTAACACCCCTCTAAGCATTTGTTTTCAGTAC

NORAD TTCCTAGGAGTGGTTGCATTTGGGAATGGAATTGTTAAAACTTGATGCTTAGGAGCGAAT
HCG11 TTCCTAGGAGTGGTTGCATTTGGGAATGGAATTGTTAAAACTTGATGCTTAGGAGCGTAT

NORAD GCAGACTATTCATTGGGTGTTTGGGGTGGGGGAAGGGGGGGTGGGCAGAGGAGGTATGCA
HCG11 GCTGACTATTCACTGCGTGG-TGGGGTGGGG-----------AGGAGGAGGAGGTATGCA

NORAD GGGAGAGGGGTTCTGTGCTCCTGAG----ATTAGTTCAGATGGTCTAACCATTGTTCTAT
HCG11 GGGAGAAGGGTTCTGTGCCCCTGAGTTAGATTAGTTCAGATGGTCTAACCATTGTTCTAT

NORAD ATGTGCATTTTA----GTTAATATTGTGTATTAAAGGATAA----GTCTTAATGCTCAAA
HCG11 GTATGCATTTTATTGTGTTAATATTGTGTATTAAAGGATAAACAAGTCTTAATGCTCAAA

NORAD GTATGTTAAAAATAGATGTAGTAAATCAGTCCCTTTGTGAATGTCCTTTTGTTAGTTTTT
HCG11 GTATGTTAAAAATAGATGTAGTGAATCAGTCCCTTTGTGAATGTCCTATTGTTAGTTTTT

NORAD AGGAAGGCCTGTCCTCTGGGAGTGACCTTTATTAGTCCACCCCTTGGAGCTAGACATCCT
HCG11 AGGAAGGCCTGTCTTCTGGGAGTGACCTTTATTAGTCCACTTCTTGGAGCTAGACGTCCT

NORAD GTACTTAGTCAC-GGGGATGGTGGAAGAGGGAGAAGAGGAAGGGTGAAGGGAAGGGCTCT
HCG11 ATACTTAGTCACTGGGGATGGTGAAAGAGGGAGAAGAGGAAGGGCGAAGGGAAGGGCTCT

NORAD TTGCTAGTATCTCCATATCTAGACGATGGTTTTAGATGATAACCACAGGTCTACAAGAGC
HCG11 TTGCTAGTATCTCCATTTCTAGAAGATGGTTTTAGATGATAACCACAGGTCTATATGAGC

NORAD GTTTTTAGTAAAGTGCCTGTGTTCATTGTGGACAAAG-TTATTATTTTGCAACATCTAAG
HCG11 ATTTTTAGTAAAGTGCCTGTGTTTATTGTGGACAGAGTTTATTATTTTGCAACATCTAAC

NORAD CTTTACGAAT----GGGGTGACAACTTATGATAAAAACTAGAGCTAGTG-----------
HCG11 CTTTATGAATATCTGAGGTGACAATGTGTGATAAAAACTAGAGCTAGTGGGCCAGGCGCG

NORAD --
HCG11 GTGGCTCATGCCTGTAATCCCAACATTTTGGGAGGCTGAGGCAGGCAGATCACAAGGTCA

NORAD --
HCG11 AGAGATCGAGACCATCCTGGTCAACATGGTGAAACCCCATCTCTACTAAAAATACAAAAA

NORAD --
HCG11 TTAGCTGGGCGTGGTGGCGCGTGCCTGTAGTCCCAGCTTCTCAGGAGGCTGAGGCAGGAG

NORAD --
HCG11 AATCACTTGAACCTGGGAGGCGGAGGTTGCAGTGAGCCAATATTGCGCCACTGCACTCCA

NORAD --AATTAG
HCG11 GCCTGGGCAACACAGCAAGACTCCATCTCAAAAAAAAAAAAAACTAGAGCTAGTGAATTAG

NORAD CCTATTTGTAAATACCTTTGTTATAATTGATAG----GATACATCTTGGACATGGAATTG
HCG11 CCAATTTGTAAATACCTTT-TTATAAGTGATAGAAAAGATGCATCTTGGACATGGAATTG

NORAD TTAAGCCACCTCTGAGCAGTGTATGTCAGGACTTGTTCATTAGGTTGGCAGCAGAGGGGC
HCG11 TTAAACCGCCTCTGAGCAGTATATGTCAGGACTTGTTCATTAGGTTGGCAGCAGAGGGGC

NORAD AGAAGGAATTATACAGGTAGAGATGTATGCAGATGTGTCCATATATGTCCATATTTACAT
HCG11 AGAAGGAAGTATACAGGGAGAGATGTATGCAGATGTGTCCATATGTGTCCATATTTACAT

NORAD TTTGAT---AGCCATTGATGTATGCATCTCTT--GGCTGTACTATAAGAACACATTAA--
HCG11 TTTGATGATAGCCATTGATGTATGCATCTCTTTTGGCTGTACTATAGGAATACATTAAGT

NORAD --TTCAATGGAAATACACTTTGCTAATATTTTAATGGTATAGATCTGCTAATGAATTCTC
HCG11 AATTCAATGGAAATATACCTTGCTAATATTATAATGGTATAGCTCTGTTAATGAATTCTC

NORAD TTAAAAACA-------TACTGTATTCTGTTGCTGTGTGTTTCATTTTAAATTGAGCATTA
HCG11 TTAGAAACATTATACTTAATGTATTCTGTTGCTGTATGTTTCATTTT-AATTGAGCATTA

NORAD AGGGAATGCAGCATTTAAATCAGAACTCTGCCAATGCTTTTATCTAGAGGCGTGTTGCCA
HCG11 AGGGAATGCAGCATTTAAATCAGAACTCTGCCAATGCTTTTATCTAGAGGCGTGTTGTCA

NORAD TTTTTGTCTTATATGAAATTTCTGTCCCAAGAAAGGCAGGATTACATCTTTTTTTTTTTT
HCG11 TTTTTGTCTTCTATGAAATTTTTGTCCCAAGAAAGGTAGGACTACA--------TTTTTT

NORAD TTTAGCAGTTTGAGTTGGTGTAGTGTATTCTTGGTTATCAGAATACTCATATAGCTTTGG
HCG11 TTTAACAGATTAAGTTGGTGTAGTGTATTCTTGTTTATCAAAATACTAATAAAGCTTTGG

NORAD GATTTTGAATTGGTAAATATTCATGATGTGTGAAAAATCATGATACATACTGTACAGTCT
HCG11 GATTTTGAATTGGTAAATATTCATGATGTGTCAAAAATCATGATACATACTGTACAATCT

NORAD   CAGTCCCATAAAATTGGATGTTGTGCCTACACACA----GGATCTAGAAGAATATGTCAA
HCG11   CAGTCCCATAAGATGGGATGTTGTGCCTACACACACACAGGACCTAGAAGAACATGTCAA

NORAD   ACTATAAACTGCTTGTGATTGTGAATGACTTTGTTCTTTGCTT---GTGTTTTTCAATTT
HCG11   ACTGTAAACTGCTTGTGATTGTGAATGACTTTGTTGTTTGCTTCTTGTGATTTTCAGTTT

NORAD   CCTATAATGCACATACTAAC-------TTTTAAAAAATAAAGGTTATTTTAAAAGCCTGT
HCG11   CCTATAATGCACATATTAACATATTAACTTTTTAAAATAAAGGTTATTTTAAAAGCCTGT

NORAD   ATTAAAAAAAAAAAAAAAAAAAAAAAAAAAAAAAAAAAAAA
HCG11   -TTAAAAAAAAAAA--------------------------
