# Supplementary material for: So alike yet so different. Differential expression of the long non-coding RNAs NORAD and HCG11 in breast cancer subtypes
Source: Genet Mol Biol. 2021 Mar 19;44(1):e20200153. doi: 10.1590/1678-4685-GMB-2020-0153 (PMC7976429; doi:10.1590/1678-4685-GMB-2020-0153)

Genetics and Molecular Biology

https://doi.org/10.1590/1678-4685-GMB-2020-0153

Supplementary Material to "So alike yet so different. Differential expression of the long non-coding RNAs NORAD and HCG11 in breast cancer subtypes."

Figure S2 - Kaplan-Meier plots representing TCGA BRCA entire cohort stratified by (A) NORAD and (B) HCG11 median expression.

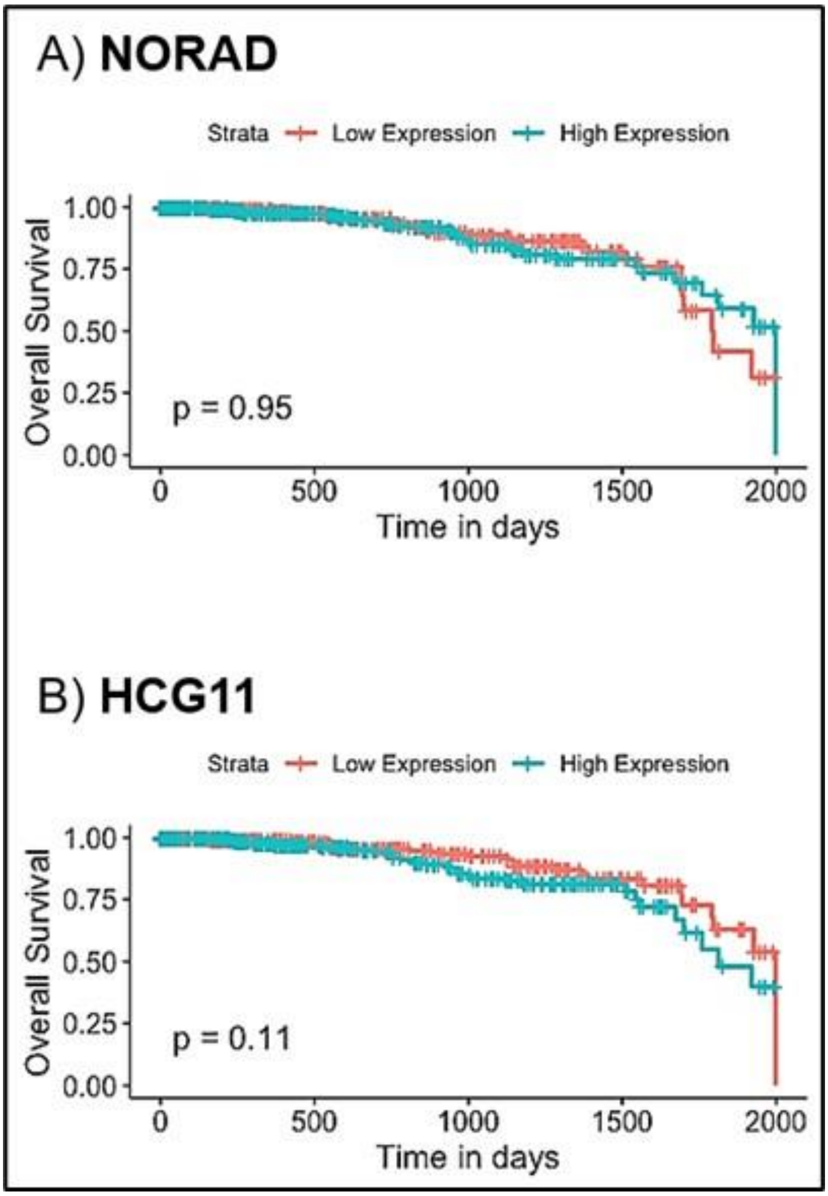

Supplement: Table S2 - Regulons reconstruction. List of genes. [file 1415-4757-GMB-44-1-e20200153-s2.pdf]
