# Supplementary material for: So alike yet so different. Differential expression of the long non-coding RNAs NORAD and HCG11 in breast cancer subtypes
Source: Genet Mol Biol. 2021 Mar 19;44(1):e20200153. doi: 10.1590/1678-4685-GMB-2020-0153 (PMC7976429; doi:10.1590/1678-4685-GMB-2020-0153)
Supplement: Figure S1 - CLUSTAL sequence alignment of NORAD and HCG11. [file 1415-4757-GMB-44-1-e20200153-s3.pdf]

Genetics and Molecular Biology

https://doi.org/10.1590/1678-4685-GMB-2020-0153

Supplementary Material to “So alike yet so different. Differential expression of the long non-coding RNAs NORAD and HCG11 in breast cancer subtypes.”

Table S1 - Clinical and histopathologic characterization of the breast tumors

Sample	Age	Diagnosis	ER	PR	ERBB2 (HER2)	Ki67 (%)	Node Metastasis
LA1	48	Invasive ductal carcinoma	POS	POS	NEG	10%	NEG
LA2	80	Invasive lobular carcinoma	POS	POS	NEG	10%	
LA3	59	Invasive ductal carcinoma	POS	POS	NEG	5-10%	NEG
LA4	57	Invasive lobular carcinoma	POS	POS	NEG	10%	POS
LA5	72	Invasive ductal carcinoma	POS	POS	NEG	5-10%	NEG
LA6	80	Mucinous carcinoma	POS	POS	NEG	10%	
LA7	71	Invasive lobular carcinoma	POS	POS	NEG	10%	NEG
LA8	55	Invasive ductal carcinoma	POS	POS	NEG	10%	NEG
LA9	65	Invasive ductal carcinoma	POS	POS	NEG	8%	NEG
LA10	32	Invasive ductal carcinoma	POS	POS	NEG	8-10%	NEG
LA11	48	Invasive ductal carcinoma	POS	POS	NEG	10%	NEG
LA12	61	Invasive ductal carcinoma	POS	POS	NEG	10%	NEG
LA13	63	Invasive ductal carcinoma	POS	POS	NEG	13%	NEG
LA14	49	Invasive ductal carcinoma	POS	POS	NEG	10-15%	NEG
LA15	85	Invasive lobular carcinoma	POS	POS	NEG	10%	NEG
LA16	52	Invasive ductal carcinoma	POS	POS	NEG	13%	
LA17	72	Invasive ductal carcinoma	POS	POS	NEG		NEG
LA18	46	Invasive ductal carcinoma	POS	POS	NEG	5-10%	POS
LA19	55	Invasive ductal carcinoma	POS	POS	NEG	10%	
LA20	74	In situ ductal carcinoma	POS	POS	NEG	10%	
LA21	45	Invasive ductal carcinoma	POS	POS	NEG	10%	NEG
TN1	39	Invasive ductal carcinoma	NEG	NEG	NEG	80%	NEG
TN2	66	Invasive ductal carcinoma	NEG	NEG	NEG	80%	NEG
TN3	72	Invasive ductal carcinoma	NEG	NEG	NEG	42%	NEG
TN4	35	Invasive ductal carcinoma	NEG	NEG	NEG	80%	
TN5	41	In situ ductal carcinoma	NEG	NEG	NEG	50%	POS
TN6	48	Invasive ductal carcinoma	NEG	NEG	NEG	80%	POS
TN7	51	Invasive ductal carcinoma	NEG	NEG	NEG	60%	NEG
TN8	74	Invasive ductal carcinoma	NEG	NEG	NEG	60%	POS
TN9	65	Invasive ductal carcinoma	NEG	NEG	NEG	90%	
TN10	53	Invasive ductal carcinoma	NEG	NEG	NEG	80%	POS
TN11	67	Invasive ductal carcinoma	NEG	NEG	NEG	80%	POS

Sample	Age	Diagnosis	ER	PR	ERBB2 (HER2)	Ki67 (%)	Node Metastasis
TN12	31	Invasive ductal carcinoma	NEG	NEG	NEG	80%	NEG
TN13	52	Invasive ductal carcinoma	NEG	NEG	NEG	95%	NEG
TN14	39	Invasive ductal carcinoma	NEG	NEG	NEG	85%	POS
TN15	48	Invasive ductal carcinoma	NEG	NEG	NEG		NEG
TN16	25	Invasive ductal carcinoma	NEG	NEG	NEG	97%	NEG
TN17	75	Invasive lobular carcinoma	NEG	NEG	NEG	13%	NEG
TN18	40	Invasive ductal carcinoma	NEG	NEG	NEG	80%	POS
TN19	50	Invasive ductal carcinoma	NEG	NEG	NEG	80%	NEG
TN20	42	Invasive ductal carcinoma	NEG	NEG	NEG	80%	POS
TN21	90	Invasive ductal carcinoma	NEG	NEG	NEG	50%	NEG
TN22	41	In situ ductal carcinoma	NEG	NEG	NEG	50%	POS
TN23	46	Invasive ductal carcinoma	NEG	NEG	NEG	10%	NEG

LA – luminal A; TN – triple-negative; IDC - Invasive ductal carcinoma; ISDC - In situ ductal carcinoma; ILC - Invasive lobular carcinoma; MC - Mucinous carcinoma; POS – positive; NEG – negative; NI – not informed.
