# Supplementary material for: So alike yet so different. Differential expression of the long non-coding RNAs NORAD and HCG11 in breast cancer subtypes
Source: Genet Mol Biol. 2021 Mar 19;44(1):e20200153. doi: 10.1590/1678-4685-GMB-2020-0153 (PMC7976429; doi:10.1590/1678-4685-GMB-2020-0153)
Supplement: Figure S2 - Kaplan-Meier plots representing TCGA BRCA entire cohort stratified by NORAD and HCG11 median expression. [file 1415-4757-GMB-44-1-e20200153-s4.pdf]

Genetics and Molecular Biology

https://doi.org/10.1590/1678-4685-GMB-2020-0153

Supplementary Material to "So alike yet so different. Differential expression of the long non-coding RNAs NORAD and HCG11 in breast cancer subtypes."

Table S2 - List of genes identified in regulons reconstruction

HCG11 Regulon		NORAD Regulon	
**Gene id**	**Gene name**	**Gene id**	**Gene name**
10257	ABCC4	91703	ACY3
84945	ABHD13	23394	ADNP
226	ALDOA	27125	AFF4
154810	AMOTL1	83607	AMMECR1L
8874	ARHGEF7	57182	ANKRD50
84938	ATG4C	139322	APOOL
23250	ATP11A	10513	APPBP2
10476	ATP5H	55198	APPL2
571	BACH1	10565	ARFGEF1
329	BIRC2	10564	ARFGEF2
54841	BIVM	94134	ARHGAP12
645	BLVRB	10079	ATP9A
662	BNIP1	8455	ATRN
11120	BTN2A1	8315	BRAP
60314	C12orf10	84280	BTBD10
54149	C21orf91	80007	C10orf88
79780	CCDC82	10944	C11orf58
219771	CCNY	90488	TMEM263
1020	CDK5	51526	OSER1
1021	CDK6	140688	NOL4L
54901	CDKAL1	25980	AAR2
84269	CHCHD5	113201	CASC4
284106	CISD3	8760	CDS2
10063	COX17	84181	CHD6
1337	COX6A1	1121	CHM
55917	CTTNBP2NL	1315	COPB1
9420	CYP7B1	51340	CRNKL1
51181	DCXR	60625	DHX35
79009	DDX50	653645	TBC1D3P1-DHX40P1
27185	DISC1	11083	DIDO1
22873	DZIP1	79982	DNAJB14
23301	EHBP1	286148	DPY19L4

HCG11 Regulon		NORAD Regulon	
**Gene id**	**Gene name**	**Gene id**	**Gene name**
2135	EXTL2	9666	DZIP3
84668	FAM126A	63916	ELMO2
57579	FAM135A	2036	EPB41L1
2195	FAT1	57669	EPB41L5
64778	FNDC3B	2059	EPS8
92579	G6PC3	56605	ERO1B
2734	GLG1	283635	FAM177A1
79158	GNPTAB	84188	FAR1
27333	GOLIM4	23014	FBXO21
10149	GPR64	26273	FBXO3
2907	GRINA	96459	FNIP1
2937	GSS	2549	GAB1
493812	HCG11	29889	GNL2
51696	HECA	2799	GNS
3315	HSPB1	51184	GPN3
653553	HSPB1P1	81488	POLR2M
3455	IFNAR2	283464	GXYLT1
3570	IL6R	11147	HHLA3
54556	ING3	83693	HSDL1
55243	KIRREL	51098	IFT52
84456	L3MBTL3	3572	IL6ST
55073	LRRC37A4P	83737	ITCH
79705	LRRK1	9778	KIAA0232
84441	MAML2	9371	KIF3B
54627	MAP10	55175	KLHL11
10150	MBNL2	54813	KLHL28
9440	MED17	84458	LCOR
84299	MIEN1	23592	LEMD3
10933	MORF4L1	92255	LMBRD2
4543	MTNR1A	647979	NORAD
340156	MYLK4	987	LRBA
4701	NDUFA7	5606	MAP2K3
29095	ORMDL2	8491	MAP4K3
132430	PABPC4L	51360	MBTPS2
5050	PAFAH1B3	4193	MDM2
9749	PHACTR2	9969	MED13
5324	PLAG1	166968	MIER3
57480	PLEKHG1	345778	MTX3
5465	PPARA	8202	NCOA3
79144	PPDPF	23054	NCOA6
55370	PPP4R1L	112597	CYTOR
5578	PRKCA	27020	NPTN
23683	PRKD3	4900	NRGN

HCG11 Regulon		NORAD Regulon	
**Gene id**	**Gene name**	**Gene id**	**Gene name**
5629	PROX1	5032	P2RY11
5727	PTCH1	55251	PCMTD2
9444	QKI	149420	PDIK1L
5911	RAP2A	51230	PHF20
9770	RASSF2	23509	POFUT1
10179	RBM7	5494	PPM1A
114822	RHPN1	11108	PRDM4
9781	RNF144A	5573	PRKAR1A
4919	ROR1	389362	PSMG4
6197	RPS6KA3	5770	PTPN1
6259	RYK	5786	PTPRA
93517	SDR42E1	22931	RAB18
26135	SERBP1	23011	RAB21
9990	SLC12A6	57403	RAB22A
1836	SLC26A2	57148	RALGAPB
4093	SMAD9	10137	RBM12
9655	SOCS5	9584	RBM39
161742	SPRED1	58490	RPRD1B
6820	SULT2B1	55291	PPP6R3
6902	TBCA	9871	SEC24D
23483	TGDS	6400	SEL1L
7046	TGFBR1	10955	SERINC3
8577	TMEFF1	84068	SLC10A7
55254	TMEM39A	29957	SLC25A24
9697	TRAM2	81031	SLC2A10
1787	TRDMT1	23443	SLC35A3
10810	WASF3	55508	SLC35E3
25937	WWTR1	80255	SLC35F5
10413	YAP1	4090	SMAD5
7539	ZFP37	144108	SPTY2D1
55888	ZKSCAN7	10254	STAM2
55205	ZNF532	6780	STAU1
ENSG00000233757.2	A2M-AS1	29966	STRN3
ENSG00000244513.2	AC022509.3	55617	TASP1
ENSG00000245105.1	AC092835.1	128637	TBC1D20
ENSG00000250899.3	AC109587.1	7057	THBS1
ENSG00000254154.4	AC125807.2	9777	TM9SF4
ENSG00000255893.1	AL121936.1	54664	TMEM106B
ENSG00000256894.1	AP000786.1	55751	TMEM184C
ENSG00000261353.1	CRYZL2P-SEC16B	84910	TMEM87B
ENSG00000213904.4	LIPE-AS1	160418	TMTC3
		112858	TP53RK
		373	TRIM23

HCG11 Regulon		NORAD Regulon	
**Gene id**	**Gene name**	**Gene id**	**Gene name**
		26133	TRPC4AP
		142940	TRUB1
		79809	TTC21B
		55293	UEVLD
		55245	UQCC1
		84669	USP32
		84640	USP38
		7716	VEZF1
		157680	VPS13B
		27072	VPS41
		11059	WWP1
		331	XIAP
		7529	YWHAB
		463	ZFHX3
		23051	ZHX3
		7559	ZNF12
		7764	ZNF217
		144348	ZNF664
		7789	ZXDA
		158586	ZXDB
		ENSG00000225733.1	FGD5-AS1
		ENSG00000233137.2	RP11-220I1.1
		ENSG00000248866.1	USP46-DT
		ENSG00000253106.1	AC090198.1
		ENSG00000260418.1	AL023284.4
		ENSG00000267104.1	TBC1D3P1-DHX40P1
		ENSG00000273472.1	AC096733.2
